# Differences in Cognitive Performance Between Individuals at Familial High Risk for Schizophrenia or Bipolar Disorder and Healthy Controls: a Systematic Review and Meta-Analysis

**DOI:** 10.1192/j.eurpsy.2025.489

**Published:** 2025-08-26

**Authors:** B. Pedruzo, C. Aymerich, A. Yorca, R. Magdaleno, A. Diaz Pons, C. Martinez, V. Ortiz, M. A. Gonzalez-Torres, R. Ayesa-Arriola, A. Catalán

**Affiliations:** 1 Department of Psychiatry, Basurto University Hospital, Bilbao; 2 Department of Psychiatry, Valdecilla Biomedical Research Institute, Santander, Spain

## Abstract

**Introduction:**

Schizophrenia and bipolar disorder are severe psychiatric disorders influenced by genetic and environmental factors, sharing clinical features, risk factors, and genetic predispositions. Cognitive impairment is a central aspect of both disorders, affecting various domains like memory, attention and executive function. Distinguishing disorder-specific cognitive impairments from those shared across psychoses is challenging. Research on Clinical High Risk (CHR) and Familial High Risk (FHR) populations is vital for identifying cognitive impairments related to vulnerability. CHR populations exhibit cognitive changes, but the evidence on FHR populations remains less clear, even though offspring of individuals with severe mental disorders face a high risk of developing these conditions.

**Objectives:**

This study aims to perform a meta-analysis of neurocognitive functioning in offspring of individuals with affective psychoes and non-affective psychoses, compared to healthy controls (HC-Off ). This meta-analysis seeks to improve statistical power of individual studies and offer more reliable estimates of cognitive deficits related to genetic vulnerability to psychotic disorders.

**Methods:**

Following PRISMA, MOOSE, and EQUATOR guidelines, a systematic literature search was conducted up to December 12th 2023. Articles were screened, and those relevant were assessed for eligibility. The inclusion criteria focused on original studies comparing neurocognitive performance between offspring of individuals with affective or non-affective psychoses and healthy controls. The primary outcome was the difference in performance across neurocognitive domains. Meta-analyses were conducted on the overall sample and separately for affective and non-affective offspring, using a random-effects model.

**Results:**

Within the analyzed domains, individuals with affected parents performed significantly worse in every domain in comparison with control group, except in Motor Functioning domain. When studied separatley, offspring of parents with affective psychosis showed significant cognitive impairments in visual and verbal learning, processing speed, and memory, with smaller deficits in other domains. In contrast, offspring of parents with non-affective psychosis exhibited more severe impairments. Social cognition and motor functioning were less affected in both groups.

**Conclusions:**

Key deficits in learning, memory, and general intelligence highlight the potential for these cognitive domains to serve as markers of vulnerability to psychosis. While both groups of offsrping show impairments, the more pronounced deficits in the non-affective group indicate a distinct cognitive risk profile. These insights may inform early interventions tailored to the specific cognitive challenges faced by high-risk populations.

**Disclosure of Interest:**

None Declared

